# Antiretroviral strategies for the treatment of pregnant HIV+ women and prevention of perinatal HIV transmission in Dodoma, Tanzania: AMANI Study

**DOI:** 10.1186/1758-2652-13-S4-P160

**Published:** 2010-11-08

**Authors:** G Liuzzi, F Vairo, Z Chaula, B Nguhuni, E Nicastri, N Bevilacqua, A Cannas, MG Paglia, G Ippolito

**Affiliations:** 1 National Institute for Infectious Diseases “L.Spallanzani”, Roma, Italy; 2 Clinical Department, Dodoma Regional Hospital, Dodoma, Tanzania; 3 Resource Center for Infectious Diseases, Dodoma Regional Hospital, Dodoma, Tanzania

## Background

The use of HAART during lactation has also been discussed as an approach to reduce postnatal HIV-1 transmission. The benefits and safety of HAART used solely for prevention of postnatal transmission in healthy HIV infected women have not yet been demonstrated in clinical trials. The extension of maternal HAART through the period of breast feeding can be proposed as pivotal strategy to reduce breast milk HIV transmission.

## Objectives

To assess the safety of breastfeeding during antiretroviral treatment in terms of virological and clinical outcomes in pregnant HIV-infected women in the Dodoma area of the United Republic of Tanzania. Secondary objective is the study of viral dynamic and therapeutic drug monitoring of antiretroviral drugs into breast milk of HIV-infected women treated with HAART during breastfeeding.

## Results

The enrolment phase of the AMANI Study started on May 2010 at Makole Health Centre (Makole UHC). After the first two months 68 HIV positive pregnant women (HPW) out of 68(100%) HCWs attending the site were counseled. 50 HPW (73.5%) were enroled in the study and are being followed up at Makole UHC. Of the remaining 16(23.5%) were not eligible and only 2(2.9%) refused to participate. Among the 50 HPW enroled only 6 (12%) have opted not to breastfeed after delivery, 12 HPW have started HAART prophylaxis from the 28 weeks of gestation, 3 HPW were found eligible and started HAART. 1 HPW has given birth on 16th July 2010 with no fetal and maternal complication. No patient reported any side effects to the ARV drugs.

## Conclusions

At the moment the study is progressing over the expectation. The procedures of the study where well accepted by the local Health Personnel with the totality of the HPWs counseled and by the HPW with only the 2.9% of refusal. Data from the study show that this kind of implementation research is well accepted by the Health personnel in terms of capacity building and by the HPW in terms of compliance and acceptance of the treatment. The benefits of the expanded treatment are well understood and overtake the discomfort of a higher pill burden and the risk of more adverse events.

